# Bacterial Glycosyltransferases: Challenges and Opportunities of a Highly Diverse Enzyme Class Toward Tailoring Natural Products

**DOI:** 10.3389/fmicb.2016.00182

**Published:** 2016-02-18

**Authors:** Jochen Schmid, Dominik Heider, Norma J. Wendel, Nadine Sperl, Volker Sieber

**Affiliations:** ^1^Chemistry of Biogenic Resources, Technische Universität MünchenStraubing, Germany; ^2^Department of Bioinformatics, Straubing Center of Science, University of Applied Sciences Weihenstephan-TriesdorfStraubing, Germany

**Keywords:** screening, bacterial glycosyltransferases, categorization of glycosyltransferases, substrate specificity, docking experiments, polysaccharide glycosyltransferases

## Abstract

The enzyme subclass of glycosyltransferases (GTs; EC 2.4) currently comprises 97 families as specified by CAZy classification. One of their important roles is in the biosynthesis of disaccharides, oligosaccharides, and polysaccharides by catalyzing the transfer of sugar moieties from activated donor molecules to other sugar molecules. In addition GTs also catalyze the transfer of sugar moieties onto aglycons, which is of great relevance for the synthesis of many high value natural products. Bacterial GTs show a higher sequence similarity in comparison to mammalian ones. Even when most GTs are poorly explored, state of the art technologies, such as protein engineering, domain swapping or computational analysis strongly enhance our understanding and utilization of these very promising classes of proteins. This perspective article will focus on bacterial GTs, especially on classification, screening and engineering strategies to alter substrate specificity. The future development in these fields as well as obstacles and challenges will be highlighted and discussed.

## Introduction

Glycosyltransferases (GTs) represent a subclass of enzymes that catalyze the synthesis of glycosidic linkages by the transfer of a sugar residue from a donor substrate to an acceptor. Acceptor substrates are mono-, di-, or oligo- carbohydrates, as well as proteins, lipids, DNA, and numerous other small molecules (Lairson et al., 2008). Therefore they play essential roles in biosynthesis pathways of oligo- and polysaccharides, as well as protein glycosylation and formation of valuable natural products (Lairson et al., 2008). Amongst the donor substrates, nucleotide-sugar conjugates represent the most prominent substrates (∼65%), but also lipid phosphate sugars and phosphate sugars are used (Ardèvol and Rovira, 2015). The mechanism for the regio- and stereo-specific transfer of the distinct sugar can occur via the inverting or retaining mechanism, which also defines the stereo-chemical outcome (α- or β-glucosides). The inverting mechanism follows a single displacement mechanism by a nucleophilic attack of the acceptor on the C-1 of the sugar donor inverting the anomeric stereochemistry. This mechanism is widely accepted and chemically elucidated (Schuman et al., 2013).

For retaining GTs different mechanisms have been proposed and the exact mechanism is still a matter of debate (Schuman et al., 2013). Latest findings based on quantum mechanics and molecular mechanics dynamic simulations indicate that two different enzyme families might have evolved, which follow either a double displacement (two S_N_2 reactions) mechanism or a front-face mechanism. One factor influencing the distinct mechanism will be the presence or absence of a putative nucleophile residue near the anomeric carbon of the donor sugar. Furthermore a competition between the front-face and double displacement mechanism was calculated by QM/MM for nucleophile-containing GTs (Rojas-Cervellera et al., 2013). The departure of the leaving group and the nucleophilic attack occur in an asynchronous manner at the same face of the glycoside (Ardèvol and Rovira, 2015).

For classification of GTs several approaches are used. The most prominent one is the classification by amino acid sequence similarities, as basically done by the Carbohydrate-Active enZYmes Database (CAZy). The CAZy database groups the different GTs into families. It comprises 97 families based on ∼215,930 entries (at 27th November, 2015). Additionally ∼4,015 sequences are not classified to this date. Nomenclature of the families is performed by use of GT and the following number of the GT family. Next to the EC 2.4 families, the CAZy classification also includes six families, belonging to EC 3.X and EC 5.X with around 395 entries. In 2012 the CAZy classification contained only ∼87,000 entries which were divided into 90 families, showing the fast development in the field of sequence identification of GTs (Gloster, 2014). In November 2015, the three families 36, 46, and 86 are still listed, but do not contain any sequences since no characterized members (GT-46) exist, or they have been deleted and merged with other GT-families (GT-36 and GT-86) based on newest findings. From the ∼215,930 listed sequences less than 1% (1,919) has been characterized. Structures are available for 161 of these, distributed over 41 families, which include solely three crystal structures, two of them for bacterial GTs. The statistical insights of the several GT-families as classified by CAZy are displayed in in **Table 1**.

**Table 1:** Statistics of CAZy database accessed at 27th November, 2015.

Fold	Mechanism	GT-Family	GTs	Characterized	Structures
GT-A	Inverting	84, 82, 43, 21, 13, 12, 7, 2	68,434 (59,527)	351 (141)	15 (7)
	Retaining	81, 78, 64, 55, 27, 24, 15, 8, 6	6,939 (3,656)	155 (30)	24 (9)
GT-B	Inverting	80, 70, 68, 65, 63, 41, 33, 30, 28, 23, 19, 10, 9, 1	32,069 (26,654)	503 (175)	52 (38) (2 crystal)
	Retaining	72, 35, 20, 5, 4, 3	65,550 (53,879)	379 (147)	38 (32) (1 crystal)
GT-C	Inverting	87, 85, 83, 66, 59, 58, 57, 50, 39, 22	8,704 (5,184)	80 (17)	8 (2)
	Retaining	–	–	–	–
Not defined	Inverting	97, 94, 92, 90, 76, 75, 74, 73, 67, 61, 56, 54, 53, 51, 49, 48, 47, 42, 40, 38, 37, 31, 29, 26, 25, 18, 17, 16, 14, 11,	28,445 (21,184)	356 (82)	16 (10)
	Retaining	96, 95, 93, 89, 88, 79, 77, 71, 69, 62, 60, 45, 44, 34, 32	5,903 (1,798)	81 (25)	7 (6)

*Numbers in clamps represent bacterial GTs*.

Use of CAZy database for classification toward substrate specificity is done by conserved three-dimensional architecture for the structures of all nucleotide sugar dependent GTs. All structures of GTs solved to date adopt one of three folds, termed GT-A, GT-B, and GT-C (Gloster, 2014). The GT-A enzymes are generally dependent on divalent metal ions and comprise two β/α/β Rossmann-like domains. A highly conserved DXD motif within the active side coordinates the metal ion, which stabilizes the charged phosphate groups of the nucleotide sugar donor, therefore supporting departure of the leaving group. The three dimensional architecture of GT-B enzymes comprises two β/α/β Rossmann-like domains that face each other. These enzymes are generally independent of metal ions, and the active site is spatially located in between the two Rossmann-like domains. The residues of the active side are involved in leaving group departure. In 2003 there was identified the third family, the GT-C enzymes, which are hydrophobic integral membrane proteins having a modified DXD signature in the first extracellular loop, and mainly using lipid phosphate linked sugar donors (Liu and Mushegian, 2003; Gloster, 2014). The limited amount of structural folds in GTs might result from the potential evolutionary origin of only few precursors’ sequences. Only GTs belonging to GT2 and GT4 are found in ancient Archaea, and it is assumed that the other families may have evolved from these two families (Coutinho et al., 2003). The next step toward increased functional prediction and substrate specificity is the classification into clans that is performed by grouping families displaying similar fold, analogous catalytic apparatus and identical mechanism (Coutinho et al., 2003; Osmani et al., 2009). These approaches will end up in so called subfamilies to further increase the functional prediction, but are also guided by the need of the researcher. Mammalian and bacterial GTs show only a very low identity on amino acid sequence level even if they synthesize the same glycan linkages (Brockhausen, 2014). Plant and bacterial GTs also have a low identity on nucleotide and amino acid level, which is the same for bacterial GTs amongst themselves, but similarities might be identified on structural level (Gloster, 2014). Next to their role in protein- and natural product glycosylation or oligo- and polysaccharide biosynthesis, they can be used for tailored chemo-enzymatic synthesis of novel, modified natural products (Luzhetskyy and Bechthold, 2008). Additionally, they play essential roles in fundamental biological processes and might be exploited for novel medical applications (Wu et al., 2012; Zhan et al., 2015). Especially by in-depth characterization and altered sugar functionality of GTs, this emerging field may develop rapidly.

## Screening for GTs Activity

Screening of GT activity is essential to characterize the GTs identified in sequenced genomes and proof the predicted activity and specificity. Within the last years several assays for GTs have been published, and were reviewed by Palcic and Sujino (2001) and Wagner and Pesnot (2010) with focus on fluorescence based methods. In general, the depletion of the acceptor or the nucleotide donor or the formation of the nucleotide or the glycosylated product can be monitored. Because of the absence of changes in the fluorescence or UV/visible spectra by GT activity, other methods to monitor substrate conversion are needed. Additionally, the assays have to be highly sensitive, caused by the low concentration of GTs in natural sources. Therefore, *radiochemical assays* represent a good choice for quantitative monitoring of GT activity by use of commercially available radiolabeled donors, and following separation of the unreacted donor from labeled reaction products. Depending on the acceptors (saccharide, protein etc.), different separation (Electrophoresis, ion exchange Chromatography, Thin layer Chromatography, organic solvent extraction) and analysis techniques (GC-MS) can be applied (Palcic and Sujino, 2001). Within the last years, several *scintillation proximity assays* have been developed to realize high throughput screening (HTS) without tedious washing steps. These assays function by immobilizing the acceptor of the glycosylation reaction on scintillation-coated microspheres, which emit a light signal, when the radio emitting labeled sugar comes in close proximity during the reaction (Hood et al., 1998; Miyashiro et al., 2005; Ahsen et al., 2008).

Non-radioactive highly sensitive assays are realized by use of *immunological methods* such as antibodies or lectins, directly identifying the reaction products (Cummings and Etzler, 2009). Mostly glycolipid acceptors are used, which have to be removed after the reaction. Within the last years several HT-methods have been developed, which adsorb the acceptor to the surface of the micro titer plate (MTP)-wells, turning the acceptor into an immobilized product, which can be specifically stained after washing steps (Lira-Navarrete et al., 2011). *Spectrophotometric assays* can be realized by coupling different enzymes. For example, NDP that is released during glycosyl transfer can react with phosphenolpyruvate (pyruvate kinase), which then releases pyruvate that can be detected by following the decrease of 340 nm for the oxidation of NADH (lactate dehydrogenase). Several adapted versions have been developed, which can be applied in high throughput and small volume also for membrane bound GTs (Brown et al., 2012). A pragmatic approach is the use of *pH-sensitive assays*. The hydrolysis of the sugar-nucleotide donor substrates into the corresponding nucleoside diphosphate results in the release of proton equivalents for all GT-catalyzed glycosylation reactions. These protons cause a color change of pH-indicators, as shown for the first time by use of phenol red (Deng and Chen, 2004) or bromothymol blue (Persson and Palcic, 2008). Another assay variant based on malachite green monitors the free phosphate (P_i_) as released from leaving nucleotides by phosphatases (Wu et al., 2011). The highly specific phosphatases do not act on sugar-nucleotides as substrate. Therefore, the concentration of released P_i_ is directly proportional to the sugar molecules transferred, enabling additional measurement of kinetic parameters. These kinds of colorimetric assays can be easily applied in HT-studies (Shen et al., 2010). *Fluorescence based methods* combine high sensitivity with operational simplicity and suitability for HTS (Gribbon and Sewing, 2003). Chemosensors with high binding selectivity toward pyrophosphate monoesters were successfully used to read out GT activity as well as inhibitor screening (Wongkongkatep et al., 2006). Assays for UTP/UDP, GDP and CMP selective fluorescent probes were developed (Chen et al., 2009) and commercial nucleotide immunodetection systems (Transcreener^TM^) are available (Lowery and Kleman-Leyer, 2006). Additionally, more and more coupled assay variants arise within the last years (Kumagai et al., 2014). The use of *mass spectrometry* (MS) obtained high impact for GT screening and characterization (Norris et al., 2001; Yang et al., 2005; Ban et al., 2012; Lauber et al., 2015). A highly sophisticated HTS approach based on immobilized oligosaccharide acceptors, placed on gold-coated islands in the geometry of a 348-well MTP exist (Ban et al., 2012). By mixing and incubating unpurified, *in vitro* expressed proteins with different sugar donors on the immobilized acceptor molecules and following MS analysis enables characterization of GT specificity as well as additional kinetic information. This example impressively shows the power of MS-based methods, to screen and characterize GTs. One of the main obstacles of GT screening and characterization approaches is the membrane localization of GTs (associated or integral). Latest findings in the field of synthetic membranes, such as nano-disks might massively enhance the functional expression and *in vitro* screening options of GTs (Inagaki et al., 2013). Additionally, optimized expression and biotransformation systems such as *Pichia Pastoris* will massively enhance the screening and characterization efficiency (Ahmad et al., 2014; Ge et al., 2014).

## Engineering of GTs

The acceptor and donor specificity in GTs reside in different well separated domains. Engineering of GT sequences is a powerful tool for altering the acceptor and donor specificity by targeting the corresponding domains. Enhanced insights into the glycosylation mechanism was obtained by structural information for, e.g., plant GTs (Wang, 2009). For the GT-A fold enzymes the N- and C-terminal domains show dissimilar architecture. The N-terminal domain consists of several β-sheets, which all are flanked by α-helical Rossmann folds and are is responsible for recognition of the sugar-nucleotide donor (Davies et al., 2005; Jank et al., 2007; Mittler et al., 2007; Erb et al., 2009). The C-terminal domain mainly contains mixed β-sheets and is responsible for the binding of the acceptor molecule. In the case of GT-B fold GTs, the N- and C -terminal domains are formed by two similar Rossmann folds and have reversed functions. The N-terminal region includes the acceptor binding site and the C-terminal domain binds the donor substrate (Erb et al., 2009). The C-terminal domains show higher similarities since they recognize the same or similar donors, whereas the N-terminal domain shows lower similarity due to the greater varieties of acceptor molecules (Wang, 2009). The C- and N-terminal domains are connected via a linker and form a cleft which acts in the case of GT-B fold containing UDP-GTs as substrate binding site. There are several examples, which describe minor mutations to significantly alter the acceptor or donor acceptance of selected GTs (Hoffmeister et al., 2001, 2002; Williams et al., 2007, 2008b). Williams and Thorson (2008) developed a fluorescence-based HTS in conjunction with error-prone PCR/saturation mutagenesis to modify proficiency and promiscuity of GTs, resulting in 200–300-fold improved enzyme activity (2008). The massively altered substrate specificity (13 different UDP-sugars) was reached by mutation of three different amino acid substituents, which were identified to serve as “hot-spots” for directed evolution. The exchange of acceptor and donor recognizing domains was described to be partly successful. In some cases it was described that swapping of larger sequence elements successfully altered the substrate specificity, whereas exchange of the whole C- or N-terminal regions might also lead to inactive versions (Kohara et al., 2007), indicating that the acceptor recognition is not strictly encoded in the single domains. Sequential domain swapping approaches can be successfully applied to identify amino acids (even single ones are described) which are responsible for the altered regioselectivity of glycosylation (Cartwright et al., 2008). For bacterial GTs several swapping experiments are described which lead to chimeric GT variants having an exchanged specificity (Fischbach et al., 2007; Hansen et al., 2009; Krauth et al., 2009; Park et al., 2009). These experiments were mainly performed with highly homologous GTs and the true modular nature of, e.g., GT-B enzymes has yet to be proved (Williams et al., 2008a). Especially in the field of bacterial GTs involved in polysaccharide production only limited information concerning substrate specificity on structural level is available (Naegeli et al., 2014). But recent activities showed enhanced possibilities to further predict the substrate and product specificity (Sánchez-Rodríguez et al., 2014).

## Structural Modeling of GTs

Difficulties with high-level expression, purification, and crystallization hampers crystal structure determinations for GTs (Breton et al., 2006). Additionally, the ratio of loops to secondary elements is high in GTs. Most of these loops have a high flexibility, therefore resulting in a low electron density, thus limiting the detailed description of the catalytic domains. Additionally, GTs show a donor substrate induced conformational change (open and closed active conformation), which mainly involves the flexible loops (Boix et al., 2001, 2002; Qasba et al., 2002; Ramakrishnan et al., 2002). The low degree of sequence similarity within most of the CAZY families renders molecular modeling difficult. Fold recognition as theoretical approach, named as “threading” (Godzik, 2003) categorizes GTs, but still had some limitations so far and needed experimental proof. Additionally, the weak scores in fold recognition of many GTs might indicate not yet identified novel folds, as recently shown, when a forth fold (GT-D) was proposed (Zhang et al., 2014). But, multivariate sequence analysis in combination with fold recognition proofed to be useful for predicting folds and mechanisms for *Escherichia coli* and *Synechocystis* GTs (Rosén et al., 2004). However, most models still have a low confidence index for flexible loops and the highly variable regions, what represent the major problem for modeling acceptor sites. Therefore it can only be applied when the target and the template have sufficient identity, to allow docking of nucleotide sugars and acceptors (Heissigerová et al., 2003). Specificity toward the sugar donor and acceptor was shown to be determined by a few critical residues in the binding site (Meech et al., 2012; Naegeli et al., 2014). Especially the flexible loops involved in GT mechanism have been the subject of studies by molecular dynamic (MD) simulation (Persson et al., 2001; Ramakrishnan and Qasba, 2001; Gunasekaran and Nussinov, 2004; Šnajdrová et al., 2004). These results demonstrate the correlated motions of several loops as well as the importance of contacts between loops in the mechanism (Breton et al., 2006). Docking of substrates is a difficult task because of the presence of phosphate and divalent cation as well as the flexibility of the nucleotide sugar, but appropriate energy parameters have been developed (Petrova et al., 1999) and latest reports show highly promising results to improve our understanding and prediction of substrate specificity of especially bacterial GTs (Zhang et al., 2014; Pandey et al., 2015; Zuegg et al., 2015).

## Future Perspectives

**FIGURE 1:**
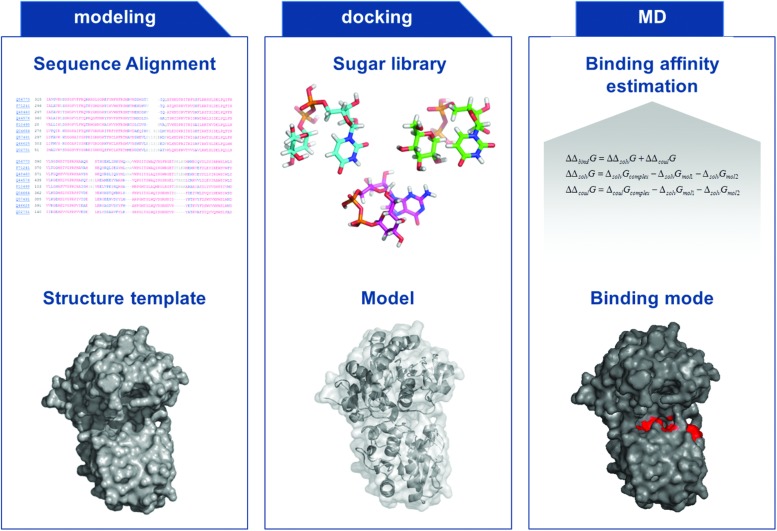
**General Flow-chart for the specific characterization of glycosyltransferases (GTs), based on optimized modeling and docking experiments in combination with final binding affinity estimation, to predict substrate specificity**.

Most computational methods make use of sequence-based comparisons for accurate prediction of substrate specificity. However, these approaches are rather limited due to the high sequence variability within the GT families. Sánchez-Rodríguez et al. (2014) used a sequence-based strategy combined with a network-based approach to infer the putative substrate classes of these predicted GTs thereby taking into account genomic organization. Due to the determination of several GT structures in the recent years (Gloster, 2014), structure-based approaches might be a promising alternative for substrate specificity prediction in the future. However, accurate ligand-protein binding affinity prediction, for a set of similar binders, is a major challenge. In general, docking calculations alone perform unsatisfactorily in these settings. But docking calculations, followed by MDs simulations and free energy calculations can be applied to improve the predictions, keeping in mind that glycosylation pattern of bacterial GTs is highly diverse and complex (e.g., rare sugars). Therefore, the transferred sugar moiety might not have been discovered yet (**Figure 1**).

A number of studies have shown that refining docking calculations by performing MD and free energy calculations starting from docked ligand positions can increase the accuracy of binding affinity predictions (Stjernschantz et al., 2006; Carlsson et al., 2008; Wünsch et al., 2012; Jiang et al., 2013). The improved accuracy of the simulations is mainly due to the increased level of molecular details, using a flexible and explicitly solvated protein. To this end, one could try to identify binding pockets on the GT structures by using docking calculations of the sugar molecules. Moreover, one could then calculate and compare the binding affinities of different sugar molecules by means of MD simulations combined with free energy calculations, e.g., with the Molecular Mechanics Poisson-Boltzmann Surface Area method (MM-PBSA) to determine substrate specificity (Kollman et al., 2000). Additionally, further approaches such as HTS crystallization and characterization (Zhu et al., 2013) of bacterial GTs are necessary to enhance our knowledge on bacterial GTs, especially involved in oligo and polysaccharide biosynthesis. Additional improved structural, modeling, and mutational studies are needed and on the way to further progress in the understanding of these highly attractive class of enzymes. These approaches in combination with novel expression systems and sophisticated tools to analyze integral membrane GTs as well as carbohydrate analysis (Rühmann et al., 2014) will enable efficient utilization of this enzyme class to efficiently tailor natural products.

## Author Contributions

JS, DH, and VS outlined the structure of the manuscript. JS and DH wrote the main parts of the manuscript. JS, NS, and NW did statistics on CAZy database and were involved in writing. All authors read and approved the final manuscript

## Conflict of Interest Statement

The authors declare that the research was conducted in the absence of any commercial or financial relationships that could be construed as a potential conflict of interest.

## References

[B1] Ahmad M. Hirz M. Pichler H. Schwab H. (2014). Protein expression in *Pichia pastoris*: recent achievements and perspectives for heterologous protein production. *Appl. Microbiol. Biotechnol.* 98 5301–5317. 10.1007/s00253-014-5732-5 24743983 PMC4047484

[B2] Ahsen O. V. Voigtmann U. Klotz M. Nifantiev N. Schottelius A. Ernst A. (2008). A miniaturized high-throughput screening assay for fucosyltransferase VII. *Anal. Biochem.* 372 96–105. 10.1016/j.ab.2007.08.029 17923099

[B3] Ardèvol A. Rovira C. (2015). Reaction mechanisms in carbohydrate-active enzymes: glycoside hydrolases and glycosyltransferases. insights from ab initio quantum mechanics/molecular mechanics dynamic simulations. *J. Am. Chem. Soc.* 137 7528–7547. 10.1021/jacs.5b01156 25970019

[B4] Ban L. Pettit N. Li L. Stuparu A. D. Cai L. Chen W. (2012). Discovery of glycosyltransferases using carbohydrate arrays and mass spectrometry. *Nat. Chem. Biol.* 8 769–773. 10.1038/nchembio.1022 22820418 PMC3471075

[B5] Boix E. Swaminathan G. J. Zhang Y. Natesh R. Brew K. Acharya K. R. (2001). Structure of UDP Complex of UDP-galactose:β-Galactoside-α-1,3-galactosyltransferase at 1.53-Å resolution reveals a conformational change in the catalytically important C terminus. *J. Biol. Chem.* 276 48608–48614. 11592969 10.1074/jbc.M108828200

[B6] Boix E. Zhang Y. Swaminathan G. J. Brew K. Acharya K. R. (2002). Structural basis of ordered binding of donor and acceptor substrates to the retaining glycosyltransferase, α-13-galactosyltransferase. *J. Biol. Chem.* 277 28310–28318. 10.1074/jbc.M202631200 12011052

[B7] Breton C. Šnajdrová L. Jeanneau C. Koča J. Imberty A. (2006). Structures and mechanisms of glycosyltransferases. *Glycobiology* 16 29R–37R. 10.1093/glycob/cwj016 16037492

[B8] Brockhausen I. (2014). Crossroads between bacterial and mammalian glycosyltransferases. *Front. Immunol.* 5:492 10.3389/fimmu.2014.00492 PMC4202792 25368613

[B9] Brown C. Leijon F. Bulone V. (2012). Radiometric and spectrophotometric in vitro assays of glycosyltransferases involved in plant cell wall carbohydrate biosynthesis. *Nat. Protoc.* 7 1634–1650. 10.1038/nprot.2012.089 22899332

[B10] Carlsson J. Boukharta L. Åqvist J. (2008). Combining docking, molecular dynamics and the linear interaction energy method to predict binding modes and affinities for non-nucleoside inhibitors to HIV-1 reverse transcriptase. *J. Med. Chem.* 51 2648–2656. 10.1021/jm7012198 18410085

[B11] Cartwright A. M. Lim E. K. Kleanthous C. Bowles D. J. (2008). A kinetic analysis of regiospecific glucosylation by two glycosyltransferases of *Arabidopsis thaliana*: domain swapping to introduce new activities. *J. Biol. Chem.* 283 15724–15731. 10.1074/jbc.M801983200 18378673 PMC3259630

[B12] Chen X. Jou M. J. Yoon J. (2009). An “Off-On” type UTP/UDP selective fluorescent probe and its application to monitor glycosylation process. *Org. Lett.* 11 2181–2184. 10.1021/ol9004849 19366258

[B13] Coutinho P. M. Deleury E. Davies G. J. Henrissat B. (2003). An evolving hierarchical family classification for glycosyltransferases. *J. Mol. Biol.* 328 307–317. 10.1016/S0022-2836(03)00307-3 12691742

[B14] Cummings R. D. Etzler M. E. (2009). *Antibodies and Lectins in Glycan Analysis*. New York, NY: Cold Spring Harbor Laboratory Press. 20301245

[B15] Davies G. J. Gloster T. M. Henrissat B. (2005). Recent structural insights into the expanding world of carbohydrate-active enzymes. *Curr. Opin. Struct. Biol.* 15 637–645. 10.1016/j.sbi.2005.10.008 16263268

[B16] Deng C. Chen R. R. (2004). A pH-sensitive assay for galactosyltransferase. *Anal. Biochem.* 330 219–226. 10.1016/j.ab.2004.03.014 15203327

[B17] Erb A. Weiß H. Härle J. Bechthold A. (2009). A bacterial glycosyltransferase gene toolbox: generation and applications. *Phytochemistry* 70 1812–1821. 10.1016/j.phytochem.2009.05.019 19559449

[B18] Fischbach M. A. Lai J. R. Roche E. D. Walsh C. T. Liu D. R. (2007). Directed evolution can rapidly improve the activity of chimeric assembly-line enzymes. *Proc. Natl. Acad. Sci. U.S.A.* 104 11951–11956. 10.1073/pnas.0705348104 17620609 PMC1924594

[B19] Ge C. Gomez-Llobregat J. Skwark M. J. Ruysschaert J. M. Wieslandg glyer A. Linden M. (2014). Membrane remodeling capacity of a vesicle-inducincosyltransferase. *FEBS J.* 281 3667–3684. 10.1111/febs.12889 24961908

[B20] Gloster T. M. (2014). Advances in understanding glycosyltransferases from a structural perspective. *Curr. Opin. Struct. Biol.* 28 131–141. 10.1016/j.sbi.2014.08.012 25240227 PMC4330554

[B21] Godzik A. (2003). Fold recognition methods. *Methods Biochem. Anal.* 44 525–546. 12647403 10.1002/0471721204.ch26

[B22] Gribbon P. Sewing A. (2003). Fluorescence readouts in HTS: no gain without pain? *Drug Discov. Today* 8 1035–1043. 10.1016/S1359-6446(03)02895-2 14690634

[B23] Gunasekaran K. Nussinov R. (2004). Modulating functional loop movements: the role of highly conserved residues in the correlated loop motions. *ChemBioChem* 5 224–230. 10.1002/cbic.200300732 14760744

[B24] Hansen E. H. Osmani S. A. Kristensen C. Møller B. L. Hansen J. (2009). Substrate specificities of family 1 UGTs gained by domain swapping. *Phytochemistry* 70 473–482. 10.1016/j.phytochem.2009.01.013 19261311

[B25] Heissigerová H. Breton C. Moravcová J. Imberty A. (2003). Molecular modeling of glycosyltransferases involved in the biosynthesis of blood group A, blood group B, Forssman, and iGb3 antigens and their interaction with substrates. *Glycobiology* 13 377–386. 10.1093/glycob/cwg042 12626391

[B26] Hoffmeister D. Ichinose K. Bechthold A. (2001). Two sequence elements of glycosyltransferases involved in urdamycin biosynthesis are responsible for substrate specificity and enzymatic activity. *Chem. Biol.* 8 557–567. 10.1016/S1074-5521(01)00039-4 11410375

[B27] Hoffmeister D. Wilkinson B. Foster G. Sidebottom P. J. Ichinose K. Bechthold A. (2002). Engineered urdamycin glycosyltransferases are broadened and altered in substrate specificity. *Chem. Biol.* 9 287–295. 10.1016/S1074-5521(02)00114-X 11927254

[B28] Hood C. M. Kelly V. A. Bird M. I. Britten C. J. (1998). Measurement of α(1-3) fucosyltransferase activity using scintillation proximity. *Anal. Biochem.* 255 8–12. 10.1006/abio.1997.2449 9448836

[B29] Inagaki S. Ghirlandob R. Grisshamme R. (2013). Biophysical characterization of membrane proteins in nanodiscs. *Methods* 59 287–300. 10.1016/j.ymeth.2012.11.006 23219517 PMC3608844

[B30] Jank T. Giesemann T. Aktories K. (2007). Rho-glucosylating *Clostridium difficile* toxins A and B: new insights into structure and function. *Glycobiology* 17 15R–22R. 10.1093/glycob/cwm004 17237138

[B31] Jiang Q.-Q. Bartsch L. Sicking W. Wich P. R. Heider D. Hoffmann D. (2013). A new approach to inhibit human [small beta]-tryptase by protein surface binding of four-armed peptide ligands with two different sets of arms. *Organ. Biomol. Chem.* 11 1631–1639. 10.1039/c3ob27302d 23358683

[B32] Kohara A. Nakajima C. Yoshida S. Muranaka T. (2007). Characterization and engineering of glycosyltransferases responsible for steroid saponin biosynthesis in Solanaceous plants. *Phytochemistry* 68 478–486. 10.1016/j.phytochem.2006.11.020 17204296

[B33] Kollman P. A. Massova I. Reyes C. Kuhn B. Huo S. Chong L. (2000). Calculating structures and free energies of complex molecules:? combining molecular mechanics and continuum models. *Acc. Chem. Res.* 33 889–897. 10.1021/ar000033j 11123888

[B34] Krauth C. Fedoryshyn M. Schleberger C. Luzhetskyy A. Bechthold A. (2009). Engineering a function into a glycosyltransferase. *Chem. Biol.* 16 28–35. 10.1016/j.chembiol.2008.12.003 19171303

[B35] Kumagai K. Kojima H. Okabe T. Nagano T. (2014). Development of a highly sensitive, high-throughput assay for glycosyltransferases using enzyme-coupled fluorescence detection. *Anal. Biochem.* 447 146–155. 10.1016/j.ab.2013.11.025 24299989

[B36] Lairson L. L. Henrissat B. Davies G. J. Withers S. G. (2008). Glycosyltransferases: structures, functions, and mechanisms. *Annu. Rev. Biochem.* 77 521–555. 10.1146/annurev.biochem.76.061005.092322 18518825

[B37] Lauber J. Handrick R. Leptihn S. Durre P. Gaisser S. (2015). Expression of the functional recombinant human glycosyltransferase GalNAcT2 in *Escherichia coli*. *Microb. Cell Fact.* 14:3 10.1186/s12934-014-0186-0 PMC4299809 25582753

[B38] Lira-Navarrete E. Valero-González J. Villanueva R. Martínez-Júlvez M. Tejero T. Merino P. (2011). Structural insights into the mechanism of protein O-Fucosylation. *PLoS ONE* 6:e25365 10.1371/journal.pone.0025365 PMC3180450 21966509

[B39] Liu J. Mushegian A. (2003). Three monophyletic superfamilies account for the majority of the known glycosyltransferases. *Protein Sci.* 12 1418–1431. 10.1110/ps.0302103 12824488 PMC2323934

[B40] Lowery R. G. Kleman-Leyer K. (2006). Transcreener: screening enzymes involved in covalent regulation. *Expert Opin. Ther. Targets* 10 179–190. 10.1517/14728222.10.1.179 16441236

[B41] Luzhetskyy A. Bechthold A. (2008). Features and applications of bacterial glycosyltransferases: current state and prospects. *Appl. Microbiol. Biotechnol.* 80 945–952. 10.1007/s00253-008-1672-2 18777021

[B42] Meech R. Rogers A. Zhuang L. Lewis B. C. Miners J. O. Mackenzie P. I. (2012). Identification of residues that confer sugar selectivity to UDP glycosyltransferase 3A (UGT3A) enzymes. *J. Biol. Chem.* 287 24122–24130. 10.1074/jbc.M112.343608 22621930 PMC3397839

[B43] Mittler M. Bechthold A. Schulz G. E. (2007). Structure and action of the C–C bond-forming glycosyltransferase UrdGT2 involved in the biosynthesis of the antibiotic urdamycin. *J. Mol. Biol.* 372 67–76. 10.1016/j.jmb.2007.06.005 17640665

[B44] Miyashiro M. Furuya S. Sugita T. (2005). A high-throughput screening system for α1–3 fucosyltransferase-VII inhibitor utilizing scintillation proximity assay. *Anal. Biochem.* 338 168–170. 10.1016/j.ab.2004.11.028 15707950

[B45] Naegeli A. Michaud G. Schubert M. Lin C.-W. Lizak C. Darbre T. (2014). Substrate specificity of cytoplasmic N-glycosyltransferase. *J. Biol. Chem.* 289 24521–24532. 10.1074/jbc.M114.579326 24962585 PMC4148877

[B46] Norris A. J. Whitelegge J. P. Faull K. F. Toyokuni T. (2001). Analysis of enzyme kinetics using electrospray ionization mass spectrometry and multiple reaction monitoring: fucosyltransferase V†. *Biochemistry* 40 3774–3779. 10.1021/bi010029v 11300757

[B47] Osmani S. A. Bak S. Møller B. L. (2009). Substrate specificity of plant UDP-dependent glycosyltransferases predicted from crystal structures and homology modeling. *Phytochemistry* 70 325–347. 10.1016/j.phytochem.2008.12.009 19217634

[B48] Palcic M. M. Sujino K. (2001). Assays for Glycosyltransferases. *Trends Glycosci. Glycotechnol.* 13 361–370. 10.4052/tigg.13.361

[B49] Pandey V. Dhar Y. Gupta P. Bag S. Atri N. Asif M. (2015). Comparative interactions of withanolides and sterols with two members of sterol glycosyltransferases from Withania somnifera. *BMC Bioinformatics* 16:120 10.1186/s12859-015-0563-7 PMC4407318 25888493

[B50] Park S.-H. Park H.-Y. Sohng J. K. Lee H. C. Liou K. Yoon Y. J. (2009). Expanding substrate specificity of GT-B fold glycosyltransferase via domain swapping and high-throughput screening. *Biotechnol. Bioeng.* 102 988–994. 10.1002/bit.22150 18985617

[B51] Persson K. Ly H. D. Dieckelmann M. Wakarchuk W. W. Withers S. G. Strynadka N. C. J. (2001). Crystal structure of the retaining galactosyltransferase LgtC from *Neisseria meningitidis* in complex with donor and acceptor sugar analogs. *Nat. Struct. Mol. Biol.* 8 166–175. 10.1038/84168 11175908

[B52] Persson M. Palcic M. M. (2008). A high-throughput pH indicator assay for screening glycosyltransferase saturation mutagenesis libraries. *Anal. Biochem.* 378 1–7. 10.1016/j.ab.2008.03.006 18405657

[B53] Petrova P. Koca J. Imberty A. (1999). Potential energy hypersurfaces of nucleotide-sugars: Ab initio calculations, force-field parametrization, and exploration of the flexibility. *J. Am. Chem. Soc.* 121 5535–5547. 10.1021/ja983854g

[B54] Qasba P. K. Ramakrishnan B. Boeggeman E. (2002). Substrate-induced conformational changes in glycosyltransferases. *Trends Biochem. Sci.* 30 53–62. 10.1016/j.tibs.2004.11.005 15653326

[B55] Ramakrishnan B. Balaji P. V. Qasba P. K. (2002). Crystal structure of β1,4-galactosyltransferase complex with UDP-Gal reveals an oligosaccharide acceptor binding site. *J. Mol. Biol.* 318 491–502. 10.1016/S0022-2836(02)00020-7 12051854

[B56] Ramakrishnan B. Qasba P. K. (2001). Crystal structure of lactose synthase reveals a large conformational change in its catalytic component, the β1,4-galactosyltransferase-I1. *J. Mol. Biol.* 310 205–218. 10.1006/jmbi.2001.4757 11419947

[B57] Rojas-Cervellera V. Ardèvol A. Boero M. Planas A. Rovira C. (2013). Formation of a covalent glycosyl–enzyme species in a retaining glycosyltransferase. *Chem. A Eur. J.* 19 14018–14023. 10.1002/chem.201302898 24108590

[B58] Rosén M. L. Edman M. Sjöström M. Wieslander Å (2004). Recognition of fold and sugar linkage for glycosyltransferases by multivariate sequence analysis. *J. Biol. Chem.* 279 38683–38692. 10.1074/jbc.M402925200 15148316

[B59] Rühmann B. Schmid J. Sieber V. (2014). Fast carbohydrate analysis via liquid chromatography coupled with ultra violet and electrospray ionization ion trap detection in 96-well format. *J. Chromatogr. A* 1350 44–50. 10.1016/j.chroma.2014.05.014 24861788

[B60] Sánchez-Rodríguez A. Tytgat H. Winderickx J. Vanderleyden J. Lebeer S. Marchal K. (2014). A network-based approach to identify substrate classes of bacterial glycosyltransferases. *BMC Genomics* 15:349 10.1186/1471-2164-15-349 PMC4039749 24885406

[B61] Schuman B. Evans S. V. Fyles T. M. (2013). Geometric attributes of retaining glycosyltransferase enzymes favor an orthogonal mechanism. *PLoS ONE* 8:e71077 10.1371/journal.pone.0071077 PMC3731257 23936487

[B62] Shen R. Wang S. Ma X. Xian J. Li J. Zhang L. (2010). An easy colorimetric assay for glycosyltransferases. *Biochemistry (Mosc)* 75 944–950. 10.1134/S0006297910070187 20673220

[B63] Šnajdrová L. Kulhánek P. Imberty A. Koča J. (2004). Molecular dynamics simulations of glycosyltransferase LgtC. *Carbohydr. Res.* 339 995–1006. 10.1016/j.carres.2003.12.024 15010307

[B64] Stjernschantz E. Marelius J. Medina C. Jacobsson M. Vermeulen N. P. E. Oostenbrink C. (2006). Are automated molecular dynamics simulations and binding free energy calculations realistic tools in lead optimization? An evaluation of the linear interaction energy (LIE) method. *J. Chem. Inform. Model.* 46 1972–1983. 10.1021/ci0601214 16995728

[B65] Wagner G. K. Pesnot T. (2010). Glycosyltransferases and their assays. *Chembiochem* 11 1939–1949. 10.1002/cbic.201000201 20672277

[B66] Wang X. (2009). Structure, mechanism and engineering of plant natural product glycosyltransferases. *FEBS Lett.* 583 3303–3309. 10.1016/j.febslet.2009.09.042 19796637

[B67] Williams G. J. Gantt R. W. Thorson J. S. (2008a). The impact of enzyme engineering upon natural product glycodiversification. *Curr. Opin. Chem. Biol.* 12 556–564. 10.1016/j.cbpa.2008.07.013 18678278 PMC4552347

[B68] Williams G. J. Goff R. D. Zhang C. Thorson J. S. (2008b). Optimizing glycosyltransferase specificity via “hot spot” saturation mutagenesis presents a catalyst for novobiocin glycorandomization. *Chem. Biol.* 15 393–401. 10.1016/j.chembiol.2008.02.017 18420146 PMC2813856

[B69] Williams G. J. Thorson J. S. (2008). A high-throughput fluorescence-based glycosyltransferase screen and its application in directed evolution. *Nat. Protoc.* 3 357–362. 10.1038/nprot.2007.538 18323806

[B70] Williams G. J. Zhang C. Thorson J. S. (2007). Expanding the promiscuity of a natural-product glycosyltransferase by directed evolution. *Nat. Chem. Biol.* 3 657–662. 10.1038/nchembio.2007.28 17828251

[B71] Wongkongkatep J. Miyahara Y. Ojida A. Hamachi I. (2006). Label-free, real-time glycosyltransferase assay based on a fluorescent artificial chemosensor. *Angew. Chem. Int. Ed. Engl.* 45 665–668. 10.1002/anie.200503107 16365842

[B72] Wu C.-Z. Jang J.-K. Woo M. Ahn J. S. Kim J. S. Hong Y.-S. (2012). Enzymatic glycosylation of nonbenzoquinone geldanamycin analogs via *Bacillus* UDP-glycosyltransferase. *Appl. Environ. Microbiol.* 78 7680–7686. 10.1128/AEM.02004-12 22923401 PMC3485733

[B73] Wu Z. L. Ethen C. M. Prather B. Machacek M. Jiang W. (2011). Universal phosphatase-coupled glycosyltransferase assay. *Glycobiology* 21 727–733. 10.1093/glycob/cwq187 21081508

[B74] Wünsch D. Fetz V. Heider D. Tenzer S. Bier C. Kunst L. (2012). Chemico-genetic strategies to inhibit the leukemic potential of threonine aspartase-1. *Blood Cancer J.* 2 e77 10.1038/bcj.2012.22 PMC3389164 22829979

[B75] Yang M. Brazier M. Edwards R. Davis B. G. (2005). High-throughput mass-spectrometry monitoring for multisubstrate enzymes: determining the kinetic parameters and catalytic activities of glycosyltransferases. *Chembiochem* 6 346–357. 10.1002/cbic.200400100 15678424

[B76] Zhan Y. Zhao F. Xie P. Zhong L. Li D. Gai Q. (2015). Mechanism of the effect of glycosyltransferase GLT8D2 on fatty liver. *Lipids Health Dis.* 14 43 10.1186/s12944-015-0040-3 PMC4425853 25952508

[B77] Zhang H. Zhu F. Yang T. Ding L. Zhou M. Li J. (2014). The highly conserved domain of unknown function 1792 has a distinct glycosyltransferase fold. *Nat. Commun.* 5:4339 10.1038/ncomms5339 PMC4352575 25023666

[B78] Zhu F. Wu R. Zhang H. Wu H. (2013). Structural and biochemical analysis of a bacterial glycosyltransferase. *Methods Mol. Biol. (Clifton, N.J.)* 1022 29–39. 10.1007/978-1-62703-465-4_3 PMC4087165 23765651

[B79] Zuegg J. Muldoon C. Adamson G. Mckeveney D. Le Thanh G. Premraj R. (2015). Carbohydrate scaffolds as glycosyltransferase inhibitors with in vivo antibacterial activity. *Nat. Commun.* 6:7719 10.1038/ncomms8719 PMC4530474 26194781

